# Adhesion and Stiffness of Detached Breast Cancer Cells In Vitro: Co-Treatment with Metformin and 2-Deoxy-d-glucose Induces Changes Related to Increased Metastatic Potential

**DOI:** 10.3390/biology10090873

**Published:** 2021-09-04

**Authors:** Špela Zemljič-Jokhadar, Gašper Kokot, Mojca Pavlin, Jure Derganc

**Affiliations:** 1 Institute of Biophysics, Faculty of Medicine, University of Ljubljana, Vrazov Trg 2, 1000 Ljubljana, Slovenia; gasper.kokot@mf.uni-lj.si (G.K.); jure.derganc@mf.uni-lj.si (J.D.); 2 Group for Nano and Biotechnological Application, Faculty of Electrical Engineering, University of Ljubljana, Vrazov Trg 2, 1000 Ljubljana, Slovenia; 3 Chair of Microprocess Engineering and Technology—COMPETE, University of Ljubljana, Večna Pot 113, 1000 Ljubljana, Slovenia

**Keywords:** metastatic potential, adhesion to epithelial cells, optical tweezers, cell deformability cytometry

## Abstract

**Simple Summary:**

The process of metastasis is one of the most destructive characteristics of cancer, yet it is still poorly understood. Formation of metastasis comprises several distinct steps: cancer cells first detach from the primary tumor and then enter the bloodstream where they circulate freely in the body and eventually adhere to vessel walls in distant organs where they can form secondary tumors. A recent study discovered that a co-treatment of two common drugs that interfere with cellular metabolism, metformin and 2-deoxy-D-glucose, induced cellular changes resembling those observed in metastasis: the drugs induced detachment of certain breast cancer cells and their proliferation in the floating state. In this study, we investigated if this treatment also induces other changes that are related to metastasis, i.e., if the detached cells are softer and if they are more prone to adhesion than control cells. The results of our in vitro experiments showed that this was indeed the case and thus indicate possible relations between metabolism and metastatic potential. While the results of this study cannot be directly projected to cancers in vivo, they present new observations that can be important for the analysis of cancer cell detachment and anchorage-independent growth.

**Abstract:**

Metastatic cancer cells can overcome detachment-induced cell death and can proliferate in anchorage-independent conditions. A recent study revealed that a co-treatment with two drugs that interfere with cell metabolism, metformin and 2-deoxy-D-glucose, promotes detachment of viable MDA-MB-231 breast cancer cells. In the present study, we analyzed if these detached viable MDA-MB-231 cells also exhibit other features related to cancer metastatic potential, i.e., if they are softer and more prone to adhere to epithelial cells. The cell mechanics of attached cells and floating cells were analyzed by optical tweezers and cell deformability cytometry, respectively. The adhesion was assessed on a confluent monolayer of HUVEC cells, with MDA-MB-231 cells either in static conditions or in a microfluidic flow. Additionally, to test if adhesion was affected by the state of the epithelial glycocalyx, HUVEC cells were treated with neuraminidase and tunicamycin. It was found that the treated MDA-MB-231 cells were more prone to adhere to HUVEC cells and that they were softer than the control, both in the floating state and after re-seeding to a substrate. The changes in the HUVEC glycocalyx, however, did not increase the adhesion potential of MDA-MB-231.

## 1. Introduction

One of the most lethal features of various cancer types is the formation of metastasis, a process where cancer cells spread from the primary tumor to other organs or tissues to form secondary tumors. The metastatic process involves several distinct steps, starting with detachment from the primary tumor and entry into the bloodstream by intravasation [1,2,3,4,5]. Some circulating tumor cells (CTCs) eventually adhere to the endothelial cells at the vessel walls at distant parts of the body and exit blood circulation by extravasation [6,7]. In a new microenvironment, they can proliferate and form secondary tumors [4]. During these distinct steps in the metastatic progression, cancer cells have to adapt to changing external conditions in a biochemical as well as in a mechanical sense (compressive stress, fluid shear stress, etc.). Unlike cancer tissues, individual cancerous cells from patients or established cell lines are softer and more deformable than normal cells [8,9,10,11], which facilitates their passage through the endothelium. Therefore, mechanical properties were explored as an additional marker of the metastatic potential.

Normal epithelial cells are anchorage dependent, and when they detach from the extracellular matrix, they enter a particular type of cell death called anoikis [12]. However, cancer cells avoid anoikis and can therefore survive in the anchorage-independent conditions during the process of metastasis formation [12,13]. One of the commonly used in vitro models of highly metastatic and tumorigenic cells are triple-negative breast cancer cells MDA-MB-231 [14]. In a study focusing on detachment of MDA-MB-231 cells, Bizjak et al. discovered that combined treatment with metformin and 2-deoxy-D-glucose (2DG) induced massive detachment of viable cells that were able to proliferate again upon re-seeding [15]. Metformin and 2DG are both drugs that are currently under investigation as anticancer agents through their effects on cellular metabolism. Metformin is a commonly used type II diabetes drug that inhibits oxidative phosphorylation, while 2DG is an inhibitor of glycolysis.

In the next step of the metastatic process, the adhesion of CTCs to endothelial cells, it is crucial that both cell types express adequate ligands and receptors [6]. An important factor for CTC adhesion is the state of the endothelial glycocalyx (GLX) [16,17], as GLX represents a selective barrier by hindering the binding between receptors on the endothelium and the ligands on cancer cells [18]. However, during many pathologic conditions, the endothelial GLX is impaired, and its components are shed into the vessel lumen, so the GLX thickness is reduced [19,20]. In healthy endothelium, the adhesion molecules are embedded within the GLX layer, but when the GLX is lost, these adhesion molecules become exposed, which increases the possibility of CTC adhesion [19]. 

As shown by Bizjak et al. [15], co-treatment of MDA-MB-231 cells with metformin and 2DG (met2DG treatment) produced a subpopulation of MDA-MB-231 cells that are prone to detach and survive in floating conditions [15], which is a prominent feature of metastatic cells. In the present work, we analyzed if this subpopulation of the floating MDA-MB-231 cells, detached by met2DG treatment, also has some other features that can be related to a higher metastatic potential. Specifically, we tested if met2DG-treated MDA-MB-231 cells are more prone to adhesion and if they are softer than normal MDA-MB-231 cells. The adhesion was measured on confluent human umbilical vein endothelial cells (HUVEC) that were additionally treated with neuraminidase and tunicamycin, which are drugs that impair the GLX on different levels. The sialic-degrading enzyme neuraminidase directly influences the GLX composition, and tunicamycin blocks N-glycosylation, which is important in the formation of GLX components. We found that the subpopulation of met2DG-treated MDA-MB-231 cells is indeed softer and more prone to adhesion, but their adhesion was not increased by the degradation of HUVEC GLX. 

## 2. Materials and Methods

### 2.1. Cell Lines and Treatments

HUVEC (ATCC) were grown in serum-reduced minimum essential medium (MEM) (Life Technologies, Carlsbad, CA, USA) supplemented with 5% fetal bovine serum (Life Technologies) and MDA-MB-231 cells (ATCC) in RPMI medium (Lonza, Basel, Switzerland) supplemented with 10% fetal bovine serum (Life Technologies) and 4.5 g/L glucose. Antibiotics (streptomycin-penicillin) (Life Technologies) were added to both media. 

To obtain floating MDA-MB-231 cells, they were first grown in a standard T-25 cell culture flask. The control MDA-MB-231 cells were detached by trypsinization 48 h after seeding and then harvested from the solution. The floating met2DG-treated MDA-MB-231 cells were treated with 5mM metformin and 0.6 mM 2-deoxy-D-glucose after seeding for 48 h and then harvested from the solution (both substances from Sigma-Aldrich, Steinheim, Germany).

For the experiments, HUVEC cells were treated one day after seeding with neuraminidase (Sigma-Aldrich) at 1 U/mL for 24 h and with tunicamycin (Sigma-Aldrich) at 5 µg/mL for 48 h. The concentrations and duration of treatments used were chosen based on the literature data and our preliminary experiments.

### 2.2. Cell Viability Assay

The cell viability was tested on control, neuraminidase and tunicamycin treated HUVEC cells. The cells were plated in 96-well microtiter plates (TPP) at a concentration of 3 × 10^4^ cells/mL. 

The cell viability was determined using the MTS (=3-(4,5-dimethylthiazol-2-yl)-5-(3-carboxymethoxyphenyl)-2-(4-sulfophenyl)-2*H*-tetrazolium) test. To each well, 20 μL of MTS (CellTiter 96 AQueous Reagent, Abcam, Cambridge, UK) was added to the cell culture medium. After 2 h, the absorbance at 490 nm was measured using a Bio-Tek microplate reader (Bio-Tek Instruments Inc., Winooski, VT, USA). The absorption corresponded to the amount of the soluble formazan product that was formed, which is directly proportional to the number of viable cells. The viability was calculated as the ratio between absorbance at 490 nm of the treated and control cells. 

### 2.3. Static Adhesion Assay

HUVEC cells were seeded (3 × 10^4^ cells/mL) into a custom-built experimental chamber with an uncoated, plasma-treated glass bottom. One day after seeding, the cells were left untreated or were treated with neuraminidase or tunicamycin. The assay was performed 48 h after seeding for control and neuraminidase treatments and 72 h for tunicamycin treatment. Floating MDA-MB-231 cells were obtained by trypsinization (control), met2DG treatment or by harvesting of cells cultured on polyHEMA (Sigma-Aldrich) coated flasks for 48 h. All MDA-MB-231 cells were stained after detachment with 0.5 µM calcein acetoxymethyl ester (Calcein AM; Life Technologies, Carlsbad, CA, USA) for 15 min in the incubator. Thereafter, 1 × 10^4^ cells/mL of stained MDA-MB-231 cells were seeded on the confluent monolayer of HUVEC cells and incubated for 20 min. Then the cells in the experimental chambers were washed thoroughly, so all weak and unattached cells were washed away. The samples were monitored under Eclipse Ti inverted microscope (Nikon, Tokyo, Japan) with epi-fluorescence, and 20 random fields of views were recorded. The adhered cells were then counted with the help of the ImageJ Cell Count Plugin [21].

### 2.4. Laminar Flow Adhesion Assay

HUVEC cells were seeded (9 × 10^4^ cells/mL) into an uncoated flow chamber (ibidi µ-slide I^0.2^ Luer). As in the static adhesion assay, the cells were whether left untreated or treated with neuraminidase or tunicamycin. For the adhesion assay, floating MDA-MB-231 cells (10^5^ cell/mL) stained with 0.5 µM calcein AM were flown over the monolayers of HUVEC cells for 15 min. The flow was kept constant using a neMESYS syringe pump (Cetoni, GmbH Korbussen, Germany) at 0.5 µL/sec. Thereafter the cells were rinsed with the growth buffer at 1 µL/sec for 10 min, so all weakly attached and unattached MDA-MB-231 cells were washed away. The samples were examined under the Eclipse Ti inverted microscope (Nikon) with epi-fluorescence. Nine fields of view from the central area of the µ-slide at 4 different distances (0.5, 1, 2 and 3 cm) from the microchannel entrance were imaged. The cells in the respective fields of views were then counted in the same way as in 2.3.

### 2.5. Optical Tweezers

The stiffness of cells was measured with optical tweezers in a custom-build PDMS experimental chamber adhered to an uncoated glass bottom. Adhered MDA-MB-231 cells were seeded into the chamber at the concentration of 1.5 × 10^4^ cells/mL. One day after seeding, met2DG solution was added to the cells for 48 h. All samples (control and treated) were measured 72 h after seeding. For the measurements of re-seeded cells, control and met2DG-treated floating MDA-MB-231 cells were seeded into the chamber at the concentration of 3 × 10^4^ cells/mL. The cells were left to attach to the substrate for one hour before the measurements were conducted. For the measurements of HUVEC cells, they were seeded into the chamber at a low concentration (1.5 × 10^4^ cells/mL) so that they did not form a confluent layer even after 24 or 48 h of treatment with neuraminidase or tunicamycin.

Cell stiffness measurements were performed on an Eclipse Ti inverted microscope (Nikon) equipped with laser tweezers (Tweez 250si, Aresis, Ljubljana, Slovenia), as described in Zemljic Jokhadar et al. [22]. In brief, the optical tweezers were set on constant optimal power (the laser wavelength was 1064 nm), and the laser beam was focused through a water immersion objective (60×, NA 1.00, Nikon) into a sample chamber. The sample heater was mounted on the microscope, and the objective was to ensure a constant temperature (37 °C). 

The experimental chamber with cells was mounted on the microscope, and silica beads with a diameter of 5.06 µm (CS01N, Bangs Labs, Fishers, IN, USA) were added into the sample, where they were trapped by optical tweezers. The beads were then positioned near the selected cell. The bead center was approximately 5 µm above the surface, which was achieved with the piezo microscope stage (Nano-LPS-200, Mad City Labs, Madison, WI, USA). By moving the piezo stage with a constant velocity of 1 µm/s, the cell was pushed into the bead. The bead position was monitored in real-time with a digital camera (Zyla 5.5, Andor, Belfast, UK) at 50 fps and by using a custom-written Matlab program (Mathworks, Natick, MA, USA). The piezo stage was automatically retracted after the bead was pushed for 0.5 µm from the center of the optical trap. The force was calculated from

F(t) = κ ∆x(t),

where κ is the stiffness of the optical trap. The deformation is calculated from the known stage position and the bead displacement:

ε(t) = x_stage (t) − ∆x(t).

The cell stiffness was determined as the slope of the linear part of the force–deformation curve (Figure S1). The indentations were small, in the range ~200 nm, and therefore probed the stiffness at the level of the cellular membrane. 

### 2.6. Deformability Cytometry

The method for deformability cytometry was adopted from Otto et al. [23]. Detached floating control MDA-MB-231 cells and floating met2DG-treated cells were centrifuged and resuspended in the viscous CellCarrier buffer (15 ± 0.8 mPa/s; Zellmechanik) to a final concentration of at least 10^6^ cells/mL. The cell suspensions were loaded into 1 mL syringe, which was fixed to the Nemesys pump (Cetoni GmbH, Korbussen, Germany) and connected to the Flic30 microfluidic chip (Zellmechanik, Dresden, Germany). The flow rate of the cell suspension was set to 0.25 µL/s, and the flow rate of the buffer was set to 0.75 µL/s [24]. Before the measurements, we waited for at least 2 min for the flow rate to stabilize. The cells were imaged with the MiniAX 200 high-speed camera (Photron) at 10,000 fps.

We processed the acquired images with custom-written image recognition software (Python 3.9.4, Wilmington, DE, USA). The automatic shape detection employed background subtraction, Gaussian blur, automatic Otsu thresholding and was filtered according to the expected size and solidity of the obtained shapes. We determined the size (projected cell surface area) *A* of the cells by counting pixels inside the automatically detected contours and the perimeter *l* as contour length. Following the definition from Otto et al. [23], the deformation of a cell was calculated as

$$1-\frac{2\sqrt{\pi A}\;}{l},$$

which yields 0 for a perfect circle. A result of a typical experiment is presented in Figure S2.

### 2.7. ELISA

MDA-MB-231 cells were seeded in uncoated or polyHEMA coated wells of a 6-well plate (TPP) at 5 × 10^4^ cells/mL. One day after seeding, cells in uncoated wells were left untreated or were treated with met2DG for 48 h. Forty-eight hours after seeding, the suspension from uncoated and polyHEMA coated wells and 72 h from wells with met2DG treated MDA-MB-231 cells was collected and centrifuged. The supernatant was stored at −80 °C and then used for ELISA analyses. For the detection of cytokine, we used immune-enzyme test Human TNF alpha Uncoated ELISA (Thermo Fisher, Waltham, MA, USA) following the manufacturer’s instructions.

### 2.8. Confocal Microscopy 

HUVEC cells were grown and treated with neuraminidase or tunicamycin as in other experiments. After the treatments, we incubated the cells with 4 µg/mL of wheat germ agglutinin, Alexafluor 488 conjugate (WGA; Thermo Fisher Scientific) in the growth media for 15 min. Then we gently washed the cells and examined the samples under the Nikon ECLIPSE TE2000-E microscope (Plan Apo TIRF objective, magnification 60×, NA = 1.45) in the confocal mode (Nikon C1). 

### 2.9. Flow Cytometry

HUVEC cells were grown and treated with neuraminidase or tunicamycin as in other experiments. HUVEC cells were seeded in 24-well culture plates for 24 h and then treated for 24 h with neuraminidase or for 48 h with tunicamycin.

After the treatments, we incubated the cells with 4 µg/mL of wheat germ agglutinin, Alexafluor 488 conjugate (WGA; Thermo Fisher Scientific) in the growth media for 15 min. Cells were washed with physiological saline and harvested by trypsinization. After trypsinization, cells were resuspended in cold PBS and measured with Attune N × T flow cytometer (Thermo Fisher Scientific). Three independent biological repeats were performed.

### 2.10. Cell Energy Phenotype Measurements

For detached cells, MDA-MB-231 cells were plated on 6-well plates at 160,000 cells per well for cells treated with 5 mM metformin + 0.6 mM 2DG for 48. For cells grown on poly-HEMA, MDA-MB-213 cells were seeded in complete RPMI medium with 1 g/L glucose on poly-HEMA coated 6-well plates at 215,000 per well for 72 h. After treatment, detached cells population were collected, and attached cells were detached with trypsin. All cell populations were spun down and resuspended in Seahorse XF RPMI 1640-based Seahorse XF Glycolytic Rate Assay medium (2 mM glutamine, 1 mM HEPES, 0 mM pyruvate, 1 g/L or 0 g/L glucose). 

The Seahorse Cell Energy Phenotype Assay (Agilent) was performed following manufacturer instructions, oxygen consumption rate (OCR) and extracellular acidification rate (ECAR) were measured using Seahorse Analyser XFp (Agilent, Santa Clara, CA, USA).

### 2.11. Statistical Analyses

For comparison of median values between the treated and the control cells, the Mann–Whitney–Wilcoxon test (Mathematica, Wolfram Research, Oxfordshire, UK) was used to calculate the *p*-values. For comparison of mean values between the treated and the control cells, the Student’s *t*-test was used. In both cases, *p*-values < 0.05 deemed significant.

## 3. Results

### 3.1. Adhesion of MDA-MB-231 Cells to a HUVEC Monolayer

**Figure 1:**
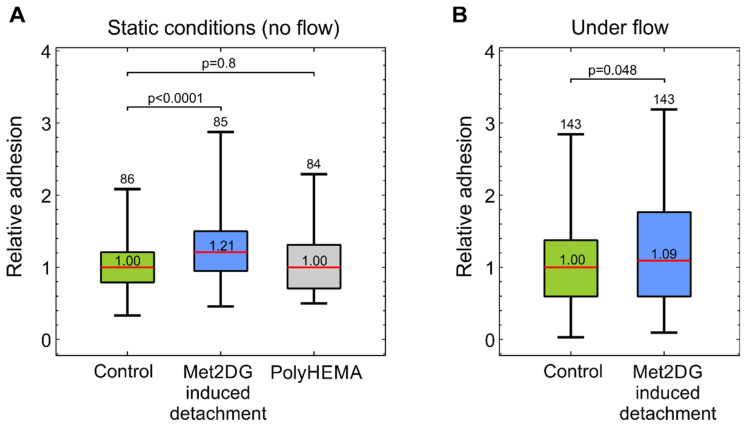
Adhesion of floating MDA-MB-231 cells onto confluent HUVEC layer measured in static conditions (**A**) and under microfluidic flow (**B**). Floating MDA-MB-231 cells were obtained by trypsinization (control, green), by met2DG treatment (blue), and by culturing the cells in polyHEMA flasks (gray). The adhesion was quantified as the number of adhered cells per field-of-view (FOV) relative to the control. The red horizontal lines represent the medians, boxes span over 50% of the data points, and the whiskers span over the total data range. The median value is indicated in the box, the number of measured FOVs is indicated at the top and the *p*-values describe the difference relative to the control. In both conditions, the met2DG-treated MDA-MB-231 cells adhered significantly more than control MDA-MB-231 cells. MDA-MB-231 cells grown on polyHEMA and tested in flow-free conditions exhibited the same adhesion as the control MDA-MB-231 cells. The results were obtained from four independent experiments.

An important step in the metastatic cascade is the adhesion of CTCs to endothelial cells. We, therefore, analyzed if floating MDA-MB-231 cells that were detached by met2DG treatment are more prone to adhere to normal HUVEC monolayer than the control MDA-MB-231 cells that were detached by the standard trypsinization protocol. The test was conducted in static conditions, as well as in a microfluidic system that mimics the shear flow in the vasculature. In the static conditions, floating MDA-MB-231 cells were placed over a HUVEC monolayer in the experimental chamber for 20 min and then thoroughly washed. In the microfluidic system, floating MDA-MB-231 cells were flown over a HUVEC monolayer in a microfluidic system for 15 min. The results showed that met2DG-treated MDA-MB-231 cells adhere significantly more to the HUVEC cells then control MDA-MB-231 cells in both the static conditions (Figure 1a) and under flow (Figure 1b). This difference was more pronounced in the static conditions (21% increase in static conditions vs. 9% increase under flow). To exclude the possibility that growth in the anchorage-independent condition is the cause of the changed adhesion potential, we also tested MDA-MB-231 cells grown in flasks coated with polyHEMA, which prevents attachment of cells. No significant difference was observed between the control MDA-MB-231 cells and MDA-MB-231 cells grown on polyHEMA coatings (Figure 1a). 

### 3.2. Stiffness of MDA-MB-231 Cells Measured by Optical Tweezers and Deformability Cytometry

We analyzed the effects of met2DG treatment on stiffness of MDA-MB-231 cells in three different conditions with two different techniques. To test if met2DG treatment alone can alter cellular stiffness, we used optical tweezers to measure the stiffness of the attached control and met2DG-treated MDA-MB-231 cells. Second, we collected floating cells obtained after 48 h of met2DG treatment and control cells detached by trypsinization and performed deformability cytometry, where deformations of individual floating cells were measured as they were pumped at high velocity through a constricted channel. To test if trypsinization has an effect on cell stiffness, we also included cells grown in flasks coated with polyHEMA. Third, we re-seeded the floating cells and probed them again with optical tweezers. Both techniques are single-cell-based, but probing with optical tweezers measures cell stiffness at small deformations, i.e., at the level of the cell membrane, while microfluidic cell deformability assay measures deformations of whole cells. 

**Figure 2:**
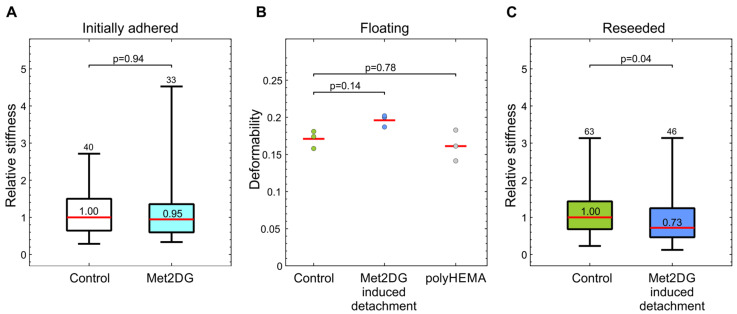
Mechanical properties of met2DG-treated MDA-MB-231 cells. (**A**) Stiffness of initially adhered MDA-MB-231 cells measured by optical tweezers. Met2DG treatment per se does not induce a significant cell softening (**B**) Deformability of floating MDA-MB-231 cells, detached by trypsinization (control), met2DG-treatment or cultured in polyHEMA coated flasks, as measured by deformability cytometry. The met2DG-treated cells are significantly more deformable. (**C**) Stiffness of re-seeded MDA-MB-231 cells measured by optical tweezers. Met2DG-treated cells are significantly softer than control cells. For measurements by optical tweezers, the cell stiffness is given relative to the control. The red horizontal lines represent the medians, boxes span over 50% of the data points, and the whiskers span over the total data range. The median value is indicated in the box, the number of measured cells indicated at the top and the *p*-values describe the difference relative to the control. For deformability cytometry, the red line represents the mean value of three independent experiments.

In the first case, we did not measure any significant difference in cell stiffness between control and met2DG-treated adhered MDA-MB-231 cells (Figure 2a). On the other hand, the floating subpopulation of met2DG-treated cells turned out to be more deformable than the floating control population, but there were no differences in deformability between control cells and the ones grown in polyHEMA coated flasks (Figure 2b). Met2DG-treated cells were also significantly less stiff (27% difference) upon re-seeding (Figure 2c).

### 3.3. Cell Energy Phenotype of met2DG-Treated MDA-MB-231 Cells

**Figure 3:**
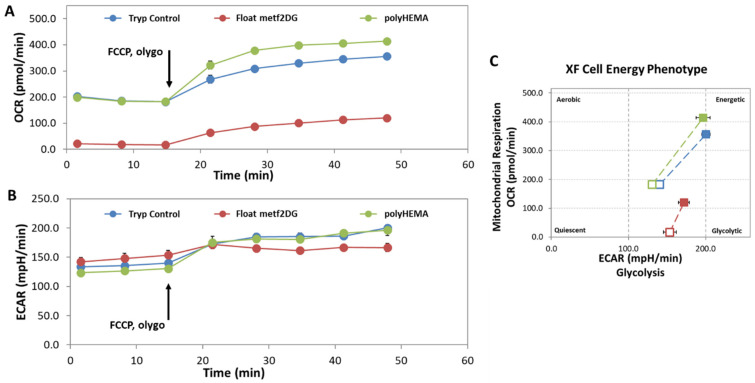
Metabolic alterations in met2DG-treated MDA-MB-231 cells. MDA-MB-231 cells were treated for 48 h with the 5 mM metformin and 0.6 mM 2DG. As a control, non-treated attached cells were harvested with trypsinization (Tryp Control) or grown on polyHEMA-coated flasks (polyHEMA). Following treatment, the cell energy phenotype of the floating met2DG treated cells (float metf2DG) was measured. (**A**) The oxygen consumption rate (OCR) and (**B**) extracellular acidification rate (ECAR) were measured with Seahorse XFp Analyzer using Cell Energy Phenotype test. The first three time points present the basal levels of OCR and ECAR; at the third time point, FCCP and oligomycin were injected. (**C**) Cell energy phenotype plot is presented for the values of OCR vs. ECAR before FCCP/oligomycin injection (open squares) and after FCCP/oligomycin injection (closed squares). Met2DG-treated cells have significantly altered cell energy phenotype with almost completely suppressed mitochondrial respiration.

As metformin and 2DG are metabolic inhibitors, we determined the cell energy phenotype of the floating met2DG-treated cells using Seahorse Analyser XFp (Figure 3). MDA-MB-231 cells were treated for 48 h with the 5 mM metformin and 0.6 mM 2DG. As a control, non-treated attached cells were harvested with trypsinization (Tryp Control) or grown on polyHEMA-coated flasks (polyHEMA). Following the treatment, the cell energy phenotype protocol was performed: The oxygen consumption rate-OCR (A) and extracellular acidification rate-ECAR (B) were measured in three time points; at the third time point, FCCP (uncoupler of mitochondrial oxidative phosphorylation) and oligomycin (inhibitor of ATP synthase) were injected. Following the injection, the time-course of OCR and ECAR in the stressed conditions were measured. (C) Cell energy phenotype plot is presented for the values of OCR vs. ECAR before FCCP/oligomycin injection (open squares) and after FCCP/oligomycin injection (closed squares). 

We observed that Met2DG-treated cells had significantly altered cell energy phenotype, with almost completely suppressed mitochondrial respiration (Figure 3a) compared to control samples, while glycolysis was only slightly increased (Figure 3b). In stressed conditions (after injections of FCCP/oligomycin), the cells in control samples (polyHEMA and tryp control) could increase the mitochondrial respiration to a higher level compared to met2DG-treated cells, which is due to the inhibitory effect of metformin on complex I of the electron transport chain. The polyHEMA and trypsinizated control samples had almost the same values in the basal conditions and were very similar in stressed conditions, thus demonstrating the cells after trypsinization have the same metabolic phenotype as the cells grown on polyHEMA. 

### 3.4. The Role of HUVEC GLX on Adhesion and Cell Stiffness of MDA-MB-231 Cells

In the second part of our study, we examined the role of epithelial GLX in the adhesion of MDA-MB-231 cells to HUVEC cells. HUVEC GLX was treated by tunicamycin, which blocks N-glycosylation, and neuraminidase, which degrades the sialic parts of GLX. As the adhesion can be affected by cell stiffness, we also measured the stiffness of treated HUVEC cells with optical tweezers.

**Figure 4:**
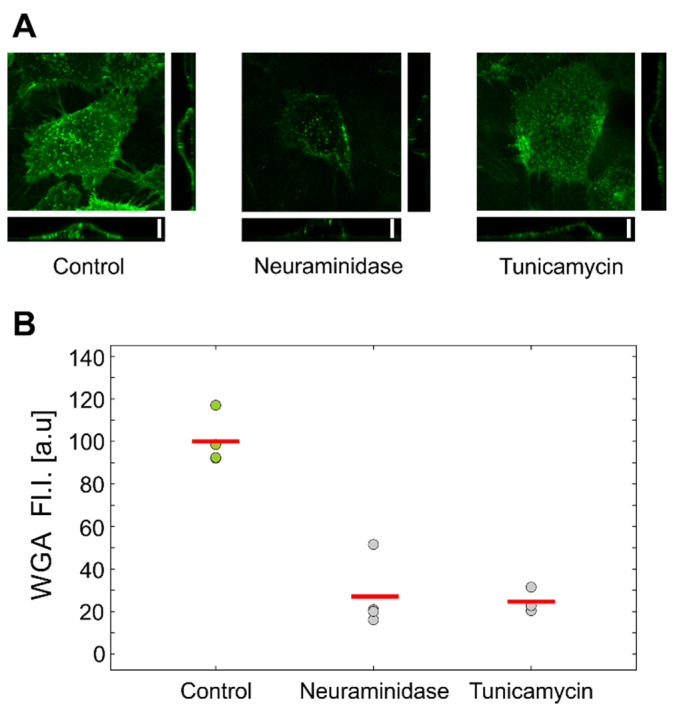
Assessment of glycocalyx in treated HUVEC cells. (**A**) Maximum intensity projection and side-projections of representative confocal images of control and treated cells. (**B**) Fluorescence intensity of WGA-stained cells obtained with flow cytometry.

We first evaluated the effects of treatments on the amount of GLX on HUVEC cells using confocal microscopy and flow cytometry by staining the cells with fluorescent WGA, which is a nonspecific GLX stain. The representative images obtained by confocal microscopy are shown in Figure 4a. The treatment with neuraminidase showed a marked decrease in fluorescent WGA signal compared to the control, while tunicamycin-treated cells showed only slightly less fluorescence. 

To quantify these results, we also analyzed the WGA-stained HUVEC cells with flow cytometry. After the treatments, the cells were harvested by trypsinization and introduced into the flow cytometer. The measured mean fluorescence intensity is presented in Figure 4b. Interestingly, the flow cytometry results showed a marked decrease in WGA intensity for both tunicamycin and neuraminidase treatments. 

**Figure 5:**
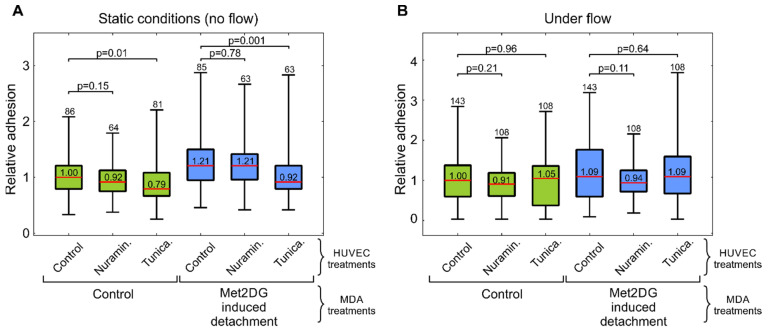
Adhesion of floating MDA-MB-231 cells onto confluent layer of treated HUVEC cells measured in flow-free conditions (**A**) and under microfluidic flow (**B**). Floating MDA-MB-231 cells were obtained by trypsinization (control) and by met2DG-treatment. HUVEC cells were either non-treated (control) or treated by neuraminidase and by tunicamycin. The adhesion was quantified as the number of adhered cells per field-of-view relative to the adhesion of non-treated MDA-MB-231 on non-treated HUVEC control. The red horizontal lines represent the medians, boxes span over 50% of the data points and the whiskers span over the total data range. The median value is indicated in the box, the number of measured FOVs indicated at the top and the *p*-values describe the difference relative to the control. Met2DG-treated MDA-MB-231 cells consistently show stronger adhesion than non-treated MDA-MB-231 cells. The results were obtained from four independent experiments.

In the next step, we analyzed if the impaired HUVEC GLX influences the adhesion of MDA-MB-231 cells. The measurements were again conducted in static conditions and under a microfluidic flow. Control MDA-MB-231 cells (detached by trypsinization) were compared to the MDA-MB-231 cells detached by met2DG treatment (Figure 5). For all HUVEC treatments, the adhesion was stronger for the met2DG-treated MDA-MB-231 cells than for control MDA-MB-231 cells. The effect of treating HUVEC cells was less prominent—the only significant difference was detected in static conditions for the treatment of HUVEC cells with tunicamycin, which reduced adhesion of control MDA-MB-231 cells by 21% and met2DG-treated MDA-MB-231 cells by 24%. Under flow, the differences between HUVEC treatments were not statistically significant.

**Figure 6:**
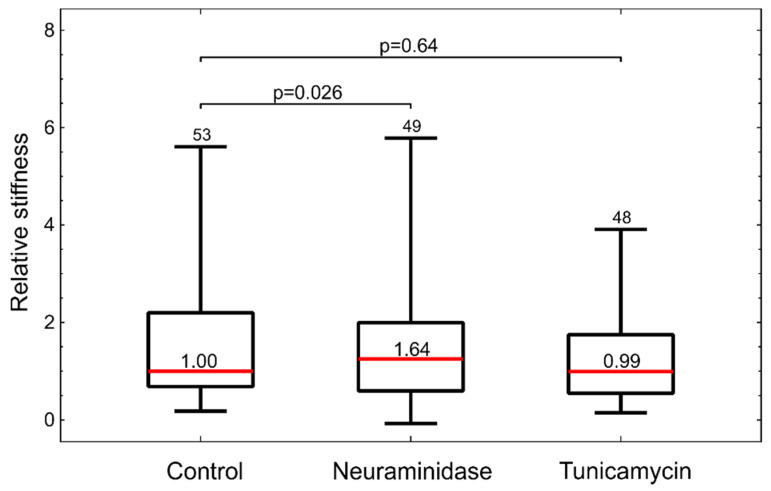
Stiffness of treated HUVEC cells measured by optical tweezers. Cell stiffness is given relative to the control. The red horizontal lines represent the medians, boxes span over 50% of the data points, and the whiskers span over the total data range. The median value is indicated in the box, the number of measured cells indicated at the top and the *p*-values describe the difference relative to the control. The results were obtained from four independent experiments.

Finally, we tested if the treatments of HUVEC cells affected their stiffness. Again we employed the measurements with optical tweezers. While tunicamycin-treated cells showed no marked changes, the neuraminidase-treated cells were significantly stiffer than the control (Figure 6).

## 4. Discussion 

Cancer cells can detach from the primary tumor, bypass anoikis and attach to the matrix of distant organs in the body, where they can form secondary tumors [2,4,13]. These processes are at the core of the metastatic cascade, which is still poorly understood. 

MDA-MB-231 human breast cancer cell line is commonly used to model late-stage breast cancer and has metastatic properties [25]. A study of MDA-MB-231 cell metabolism unexpectedly discovered that a co-treatment with two commonly used metabolic inhibitors and AMP-activated protein kinase activators, metformin and 2DG, resulted in a more than 10-fold increase in the number of detached, viable, floating MDA-MB-231 cells [15]. 

Since deregulation of anoikis is linked to metastatic ability, our goal was to investigate if this subpopulation of met2DG-treated, viable, floating MDA-MB-231 cells has mechanical characteristics that are related to increased metastatic potential; specifically, we analyzed their stiffness and ability to adhere to endothelial monolayer. Additionally, we analyzed the effects of two drugs that disrupt epithelial GLX on MDA-MB-231 adhesion. 

In the first part of the study, we compared the adhesion of normal and met2DG-treated MDA-MB-231 cells to normal endothelial HUVEC cells. Since the adhesion of CTC to vessel walls is most prominent in post-capillary venules, where only low shear stress is present [19,26], we conducted the adhesion assay in static, flow-free conditions, as well as in a microfluidic system that mimics physiological conditions. The flow velocity near the HUVEC monolayer in the microfluidic system reached approximately 120–220 µm/s, which was in the range observed to be favorable for cell adhesion to endothelial cells [27]. In both cases, we found that met2DG-treated cells were more prone to adhere to HUVEC monolayer than non-treated MDA-MB-231 cells (Figure 1). Under flow, this difference was not as large as in the case of the static condition (1.2×), but the adhesion was still higher (1.09×) compared to the control. Cells grown on polyHEMA coated glass under static conditions had the same relative adhesion as control cells obtained by trypsinization, which showed that the observed differences were indeed a consequence of met2DG treatment. It was also interesting to observe that in a relatively short time (the cells were flown over the monolayer for 15 min), the cells not only stopped but also adhered firmly enough to resist washing away, which confirmed that fast but weak adhesion could stabilize quickly [7]. 

Several studies had shown that cancerous cells exhibit different mechanical properties than normal cells of the same tissue origin. For example, highly invasive malignant MDA-MB-231 cells are softer than benign human mammary gland cell line MCF-10 or MCF-7, which is a non-invasive malignant breast cancer cell line [28,29,30]. The mechanical differences are present not just among normal and malignant cells but also among the cells with different metastatic potential [31]. We, therefore, compared the mechanical properties of met2DG-treated and control MDA-MB-231 cells. To analyze the cells in attached and floating conditions, we employed two different techniques: attached cells were analyzed by mechanical indentation with optical tweezers, and floating cells were analyzed by deformability cytometry. In this way, we assessed not only the differences between the treated and control cells but also if the relation in the cell stiffness between the two cell populations remained the same under different conditions. Our results showed that the treatment with met2DG alone had no effect on the stiffness of adhered MDA-MB-231 cells (Figure 2a). On the contrary, MDA-MB-231 cells that detached due to met2DG treatment were found more deformable in suspension (Figure 2b) and continued to be significantly softer even after re-seeding (Figure 2c). The treatment with met2DG thus induces detachment of a subpopulation of MDA-MB-231 cells, which retains the mechanical changes even after reattachment, i.e., in an altered mechanical environment. Note that results obtained by different measuring systems can only be used for a qualitative comparison of cell stiffness because they yield different parameters, which cannot be compared directly. Namely, optical tweezers measure stiffness at small deformations, and the microfluidic technique measures the deformability of whole cells [32]. Both methods were shown to detect mechanical changes induced by actin-disrupting Cytochalasin-D treatment [22,23], and therefore the actin cortex is the prime suspect responsible for the observed changes in MDA-MB-231 cells. For further verification, the cells will also have to be analyzed with additional methods, e.g., with AFM. 

In parallel, we observed significantly altered cell energy phenotype of the floating met2DG-treated compared to control (polyHEMA, trypsynized) MDA-MB-231 cells, with almost completely blocked mitochondrial respiration (Figure 3). On the other hand, floating control and met2DG treated cells have very similar glycolysis rate and mitochondrial respiration when compared to the attached cells, which was also determined in our previous study (Repas et al., Scientific Reports in revision). The metabolic alterations can affect several signaling pathways, among other also integrin expression, which could, in turn, affect the process of detachment and cells stiffness. 

GLX covers the endothelium, and in normal conditions, it forms a selective barrier between the endothelium and its environment by preventing adverse ligands the access to adhesion receptors. Therefore, in the second part of our study, we focused on the role of the endothelial GLX in the adhesion of MDA-MB-231 cells. We treated the HUVEC cells with two substances that affect the GLX on different levels. The endothelial GLX is composed of different glycoproteins, soluble proteins and residual chains, among others such as sialic acid, which are attached to core proteins [18]. Sialic acid can hide the recognition sites on the cell surface, but it can also act as a biological recognition site for a variety of molecules such as hormones, lectins, antibodies and inorganic cations itself [33]. Neuraminidase is a sialic acid degrading enzyme, so by applying it to endothelial cells, we directly modified the GLX composition. The second compound we used to modify the GLX was tunicamycin, an inhibitor of N-glycosylation which therefore impairs the process of glycoprotein formation. Glycosylation is a major post-translational modification and plays a crucial role in the folding, stability, subcellular localization and biological functions of glycoproteins [34]. Tunicamycin can lead to a decreased cell viability, especially in cancerous cells [35,36], and we indeed observed that the viability of HUVEC cells dropped by approximately 12% compared to control (Figure S3). 

Flow-cytometric analysis of HUVEC cells labeled with fluorescent WGA confirmed that both treatments reduced the amount of GLX (Figure 4). Interestingly, the inspection by confocal microscopy showed the reduction in GLX only for neuraminidase treatment. This correlated with the measurements by optical tweezers, which showed an increased stiffness only for the neuraminidase-treated cells, possibly due to the loss of the soft GLX layer (Figure 6). At present, we do not have a firm explanation for the observed difference between the results obtained by confocal microscopy and flow cytometry. One of the potential important factors is the difference between the techniques for GLX abundance determining: in confocal microscopy experiments, cells are attached, while in flow cytometry, cells had to be detached for analysis. 

Treatments of HUVEC cells with neuraminidase and tunicamycin did not result in an increase in the adhesion of MDA-MB-231 cells (Figure 5). In fact, the tunicamycin treatment even resulted in a slightly impaired adhesion under static conditions. This was possibly due to the wide-ranging effects of tunicamycin on cells, which were also reflected in the reduced cell viability (Figure S3). The results of the second part of our study thus showed that the GLX reduction in HUVEC cells alone was not enough to significantly increase the adhesion of MDA-MB-231 cells. This was likely the case because HUVEC cells were not activated by environmental factors such as cell-secreted cytokines, such as TNF-α, that trigger E-selectin expression [37]. Indeed, we analyzed if MDA-MB-231 cells secrete TNF-α cytokine in response to treatments with met2DG but did not find any significant difference between the treated and control cells (Figure S4). Clearly, further studies are needed to elucidate the underlying mechanisms of adhesion of MDA-MB-231 cells to the epithelium. 

## 5. Conclusions

We found that MDA-MB-231 cells that detach in vitro due to a combined treatment of two cell metabolism-interfering drugs, metformin and 2DG, are more prone to adhere to endothelial HUVEC cells than control MDA-MB-231 cells. They are also softer (more deformable) than the control cells both in suspension and after they re-attach to the glass substrate. While these features could be related to a higher metastatic potential, further studies are needed to confirm this connection in vivo. 

## Data Availability

The data presented in this study are available on request from the corresponding authors.

## Supplementary Materials

The following are available online at https://www.mdpi.com/article/10.3390/biology10090873/s1, Figure S1. A sample force–deformation curve. The cell stiffness was determined as the slope (red dashed line) of the linear part of the curve during the pushing phase. Figure S2. Deformability cytometry. (A) A representative image of a cell deformed in the fast flow in the constricted microfluidic channel (top) and the corresponding detected contour (bottom). (B) Distribution of the deformation of the cells versus their size for control and met2DG-treated MDA-MB-231 cells. The outliers are mainly false positives of the background from the detection algorithm or small cells with an insufficient contrast to faithfully detect the contour. In order to minimize the influence of outliers, the calculated mean values were taken inside the region indicated by a red ellipse, yielding >2400 unique cells for each experiment. Figure S3. Cell viability of control and treated HUVEC cells as assessed by MTS test. Cells treated by neuraminidase were measured 48 h post-seeding, and cells treated by tunicamycin were measured 72 h post-seeding. In both cases, the result was normalized to the corresponding non-treated control. The results were obtained from three independent experiments. Figure S4. TNF-alpha secretion of MDA-MB-231 cells. For detection of the cytokine, we used immune-enzyme test Human TNF alpha Uncoated ELISA. Note that all the results are in the vicinity of the detection threshold for this test.
