# Supplementary figures and images for: Adhesion and Stiffness of Detached Breast Cancer Cells In Vitro: Co-Treatment with Metformin and 2-Deoxy-d-glucose Induces Changes Related to Increased Metastatic Potential
Source: Biology (Basel). 2021 Sep 4;10(9):873. doi: 10.3390/biology10090873 (PMC8465291; doi:10.3390/biology10090873)

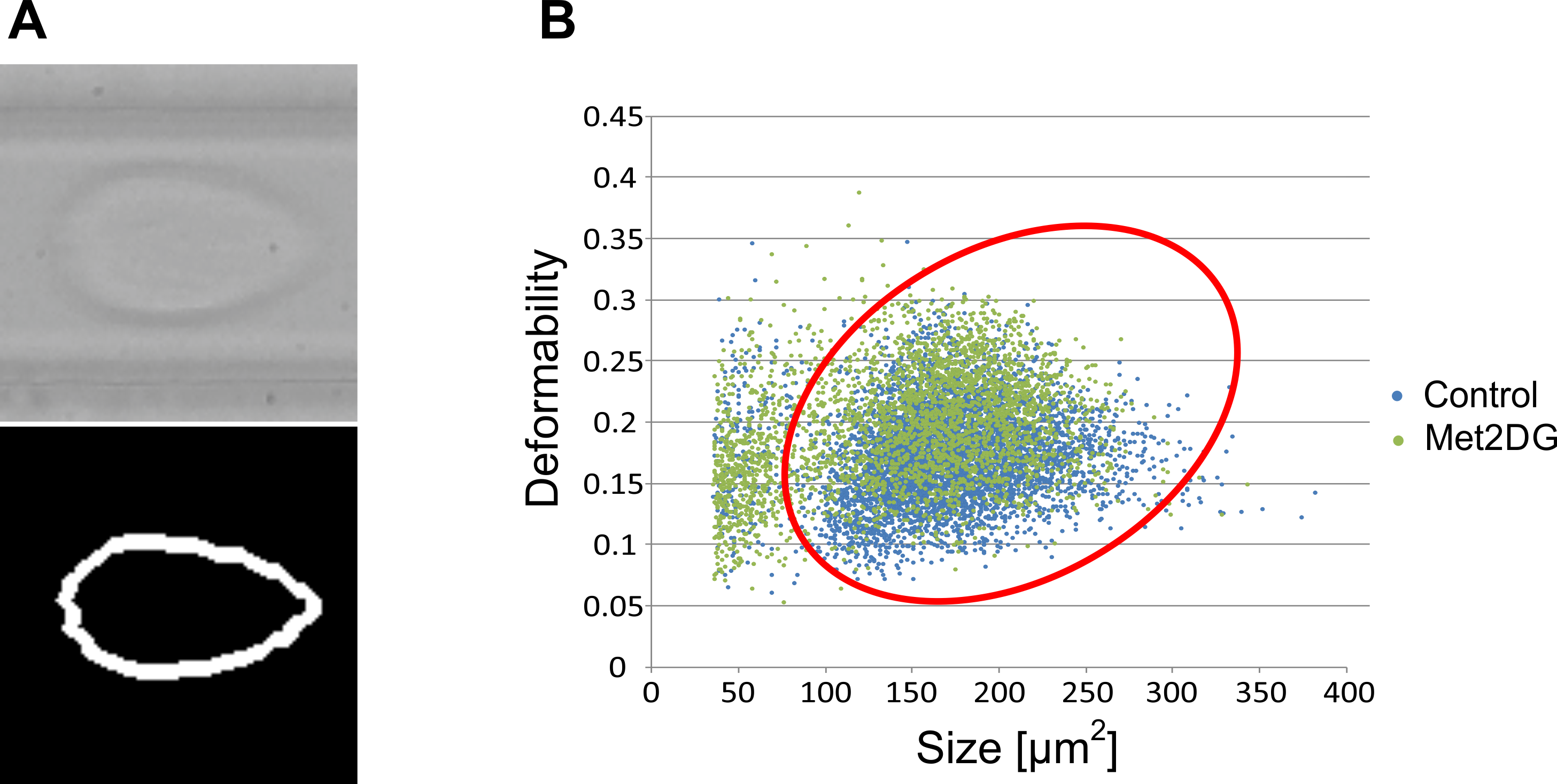

Supplement: Supplementary file 1 [file biology-10-00873-s001.zip › FigureS1.tif]

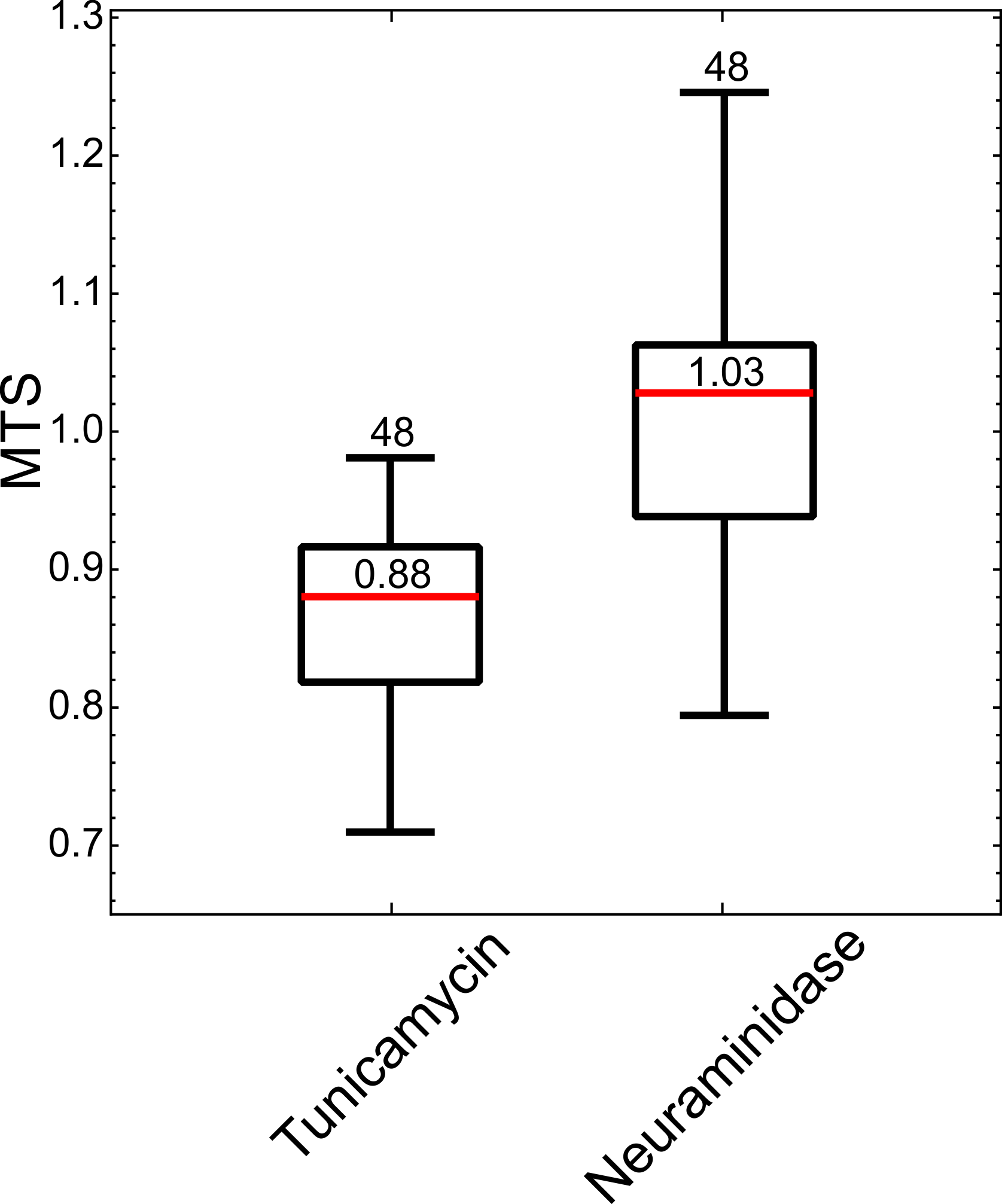

Supplement: Supplementary file 1 [file biology-10-00873-s001.zip › FigureS2.tif]

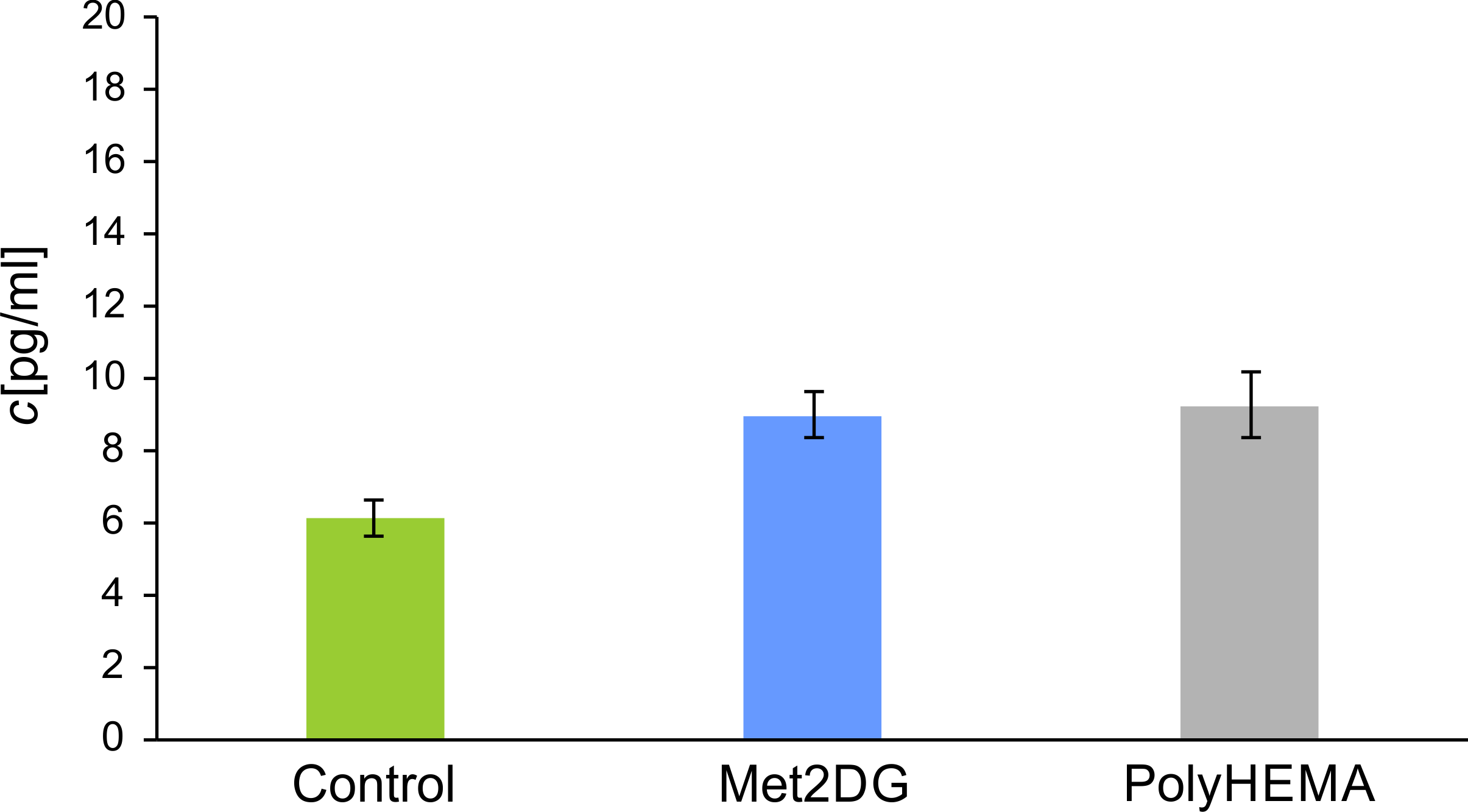

Supplement: Supplementary file 1 [file biology-10-00873-s001.zip › FigureS3.tif]
